# Guanidinium chloride–18-crown-6 (2/1)

**DOI:** 10.1107/S1600536812016959

**Published:** 2012-04-21

**Authors:** Bin Wei

**Affiliations:** a Ordered Matter Science Research Center, Southeast University, Nanjing 211189, People’s Republic of China

## Abstract

In the crystal of the title compound, 2CH_6_N_3_^+^·2Cl^−^·C_12_H_24_O_6_, the 18-crown-6 mol­ecule is located across an inversion center. The guanidinium cation links to the 18-crown-6 mol­ecule and chloride anion *via* N—H⋯O and N—H⋯Cl hydrogen bonds.

## Related literature

For applications of crown ethers, see: Clark *et al.* 1998 ▶). For ferroelectric metal-organic 18-crown-6 clathrates, see: Fu *et al.* (2009 ▶, 2011 ▶); Ye *et al.* (2006 ▶); Zhang *et al.* (2008 ▶, 2010 ▶). 
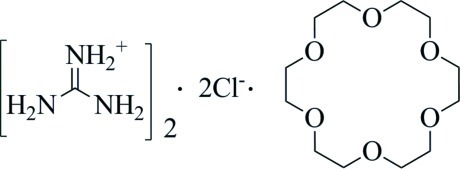


## Experimental

### 

#### Crystal data

2CH_6_N_3_^+^·2Cl^−^·C_12_H_22_O_6_
*M* *_r_* = 453.37
Monoclinic, 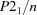

*a* = 8.9685 (18) Å
*b* = 9.7305 (19) Å
*c* = 13.995 (3) Å
β = 102.14 (3)°
*V* = 1194.0 (4) Å^3^
*Z* = 2
Mo *K*α radiation
μ = 0.31 mm^−1^
*T* = 293 K
0.20 × 0.20 × 0.20 mm

#### Data collection

Rigaku SCXmini diffractometer
Absorption correction: multi-scan (*CrystalClear*; Rigaku, 2005 ▶) *T*_min_ = 0.939, *T*_max_ = 0.940
12074 measured reflections
2732 independent reflections
1154 reflections with *I* > 2σ(*I*)
*R*_int_ = 0.130
2 standard reflections every 150 reflections intensity decay: ?

#### Refinement

*R*[*F* ^2^ > 2σ(*F* ^2^)] = 0.074
*wR*(*F* ^2^) = 0.220
*S* = 1.01
2732 reflections
127 parameters
H-atom parameters constrained
Δρ_max_ = 0.35 e Å^−3^
Δρ_min_ = −0.23 e Å^−3^

### 

Data collection: *CrystalClear* (Rigaku, 2005 ▶); cell refinement: *CrystalClear*; data reduction: *CrystalClear*; program(s) used to solve structure: *SHELXTL* (Sheldrick, 2008 ▶); program(s) used to refine structure: *SHELXTL*; molecular graphics: *SHELXTL*; software used to prepare material for publication: *SHELXTL*.

## Supplementary Material

Crystal structure: contains datablock(s) I, global. DOI: 10.1107/S1600536812016959/xu5505sup1.cif

Structure factors: contains datablock(s) I. DOI: 10.1107/S1600536812016959/xu5505Isup2.hkl

Additional supplementary materials:  crystallographic information; 3D view; checkCIF report

## Figures and Tables

**Table 1:** Hydrogen-bond geometry (Å, °)

*D*—H⋯*A*	*D*—H	H⋯*A*	*D*⋯*A*	*D*—H⋯*A*
N1—H1*A*⋯O1	0.86	2.19	2.976 (5)	152
N1—H1*B*⋯Cl1	0.86	2.49	3.294 (4)	155
N2—H2*A*⋯O1	0.86	2.36	3.102 (5)	145
N3—H3*A*⋯Cl1^i^	0.86	2.42	3.228 (4)	158

Symmetry code: (i) 
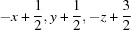
.

**Fig. 1.**
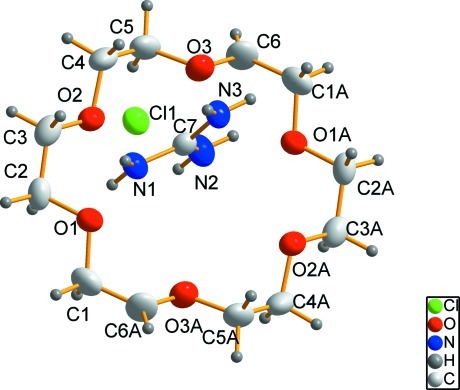
The molecular structure of the title compound, with the atomic numbering scheme. Displacement ellipsoids are drawn at the 30% probability level.

**Fig. 2.**
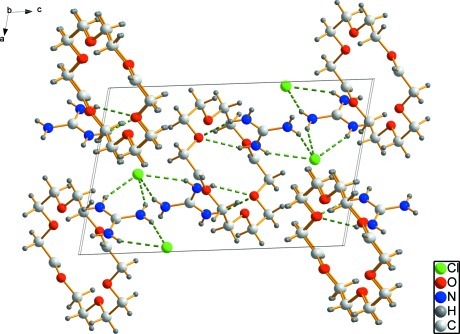
A view of the packing of the title compound, stacking along the b axis. Dashed lines indicate hydrogen bonds.

## supplementary crystallographic information

### Comment

Recent years, crown ethers have attracted much attention because of their wide application in catalysis, solvent extraction, isotopeseparation, bionice, host–guest chemistry and supramolecular chemistry (Clark *et al.*, 1998). Several 18-crown-6 clathrates were discovered to be dielectric-ferroelectric materials (Fu *et al.*, 2011), hence we design the title compound to find new hydrogen bonding type dielectric materials. Dielectric-ferroelectric materials, comprising organic ligands, metal-organic coordination compounds and organic-inorganic hybrids almost show dielectric constant of temperature-dependent (Fu *et al.*, 2009; Zhang *et al.*, 2010; Zhang *et al.*, 2008; Ye *et al.*, 2006). Unfortunately, the dielectric constant of the title compound as a function of temperature indicates that the permittivity is basically temperature-independent, below the melting point (395k-396k) of the compound, we have found that title compound has no dielectric disuniform from 80 K to 405 K. Herein we descibe the crystal structure of this compound.

At home temperature (25°C), the single-crystal X-ray diffraction reveals that the structure get crystallization in the monoclinic system, space group P 21/n and the asymmetric unit of the title compound consists of a guanidinium cation, a chloride anion and a 18-crown-6 molecule (Fig. 1). The three –NH_2_^+^ groups form guanidinium interact with a O atoms of one crown ether molecule and Cl anions through two N—H···O an two N—H···Cl hydraogen bonds (Table 1), composing a tree-dimensional crystal structure (Fig. 2).

### Experimental

The hydrochloric acid (0.36 g, 10 mmol) and guanidinium carbonate (0.9 g, 5 mmol) were dissolved in 30 ml water, and the solution was combined with methanol solution of 18-crown-6 (10 mmol). The mixture solution was stirred for 30 min to reaction fully. Blocky single crystals were obtained by slow evaporation of the filtrate after two weeks (yield 63%).

### Refinement

H atoms were placed in geometrically idealized positions with C—H = 0.97 and N—H = 0.86 Å, and refined in riding mode with U_iso_(H) = 1.2U_iso_(N,C).

### Crystal data

**Table:**

2CH_6_N_3_^+^·2Cl^−^·C_12_H_22_O_6_	*F*(000) = 484
*M**_r_* = 453.37	*D*_x_ = 1.261 Mg m^−^^3^
Monoclinic, *P*2_1_/*n*	Mo *K*α radiation, λ = 0.71073 Å
Hall symbol: -P 2yn	Cell parameters from 3638 reflections
*a* = 8.9685 (18) Å	θ = 3.0–27.5°
*b* = 9.7305 (19) Å	µ = 0.31 mm^−^^1^
*c* = 13.995 (3) Å	*T* = 293 K
β = 102.14 (3)°	Block, colorless
*V* = 1194.0 (4) Å^3^	0.20 × 0.20 × 0.20 mm
*Z* = 2	

### Data collection

**Table:**

Rigaku SCXmini diffractometer	1154 reflections with *I* > 2σ(*I*)
Radiation source: fine-focus sealed tube	*R*_int_ = 0.130
Graphite monochromator	θ_max_ = 27.5°, θ_min_ = 3.0°
ω scans	*h* = −11→11
Absorption correction: multi-scan (*CrystalClear*; Rigaku, 2005)	*k* = −12→12
*T*_min_ = 0.939, *T*_max_ = 0.940	*l* = −18→18
12074 measured reflections	2 standard reflections every 150 reflections
2732 independent reflections	

### Refinement

**Table:**

Refinement on *F*^2^	Primary atom site location: structure-invariant direct methods
Least-squares matrix: full	Secondary atom site location: difference Fourier map
*R*[*F*^2^ > 2σ(*F*^2^)] = 0.074	Hydrogen site location: inferred from neighbouring sites
*wR*(*F*^2^) = 0.220	H-atom parameters constrained
*S* = 1.01	*w* = 1/[σ^2^(*F*_o_^2^) + (0.0875*P*)^2^ + 0.3032*P*] where *P* = (*F*_o_^2^ + 2*F*_c_^2^)/3
2732 reflections	(Δ/σ)_max_ < 0.001
127 parameters	Δρ_max_ = 0.35 e Å^−^^3^
0 restraints	Δρ_min_ = −0.23 e Å^−^^3^

### Special details

**Table:**

Geometry. All e.s.d.'s (except the e.s.d. in the dihedral angle between two l.s. planes) are estimated using the full covariance matrix. The cell e.s.d.'s are taken into account individually in the estimation of e.s.d.'s in distances, angles and torsion angles; correlations between e.s.d.'s in cell parameters are only used when they are defined by crystal symmetry. An approximate (isotropic) treatment of cell e.s.d.'s is used for estimating e.s.d.'s involving l.s. planes.
Refinement. Refinement of *F*^2^ against ALL reflections. The weighted *R*-factor *wR* and goodness of fit *S* are based on *F*^2^, conventional *R*-factors *R* are based on *F*, with *F* set to zero for negative *F*^2^. The threshold expression of *F*^2^ > σ(*F*^2^) is used only for calculating *R*-factors(gt) *etc*. and is not relevant to the choice of reflections for refinement. *R*-factors based on *F*^2^ are statistically about twice as large as those based on *F*, and *R*- factors based on ALL data will be even larger.

### Fractional atomic coordinates and isotropic or equivalent isotropic displacement parameters (Å^2^)

**Table:**

	*x*	*y*	*z*	*U*_iso_*/*U*_eq_	
Cl1	0.02647 (14)	−0.02938 (12)	0.66801 (8)	0.0671 (5)	
O1	0.3181 (3)	0.2939 (3)	0.3760 (2)	0.0642 (9)	
O2	0.2159 (3)	0.5624 (3)	0.3980 (2)	0.0708 (10)	
O3	0.3634 (4)	0.7298 (3)	0.5591 (3)	0.0813 (11)	
N1	0.2175 (4)	0.1712 (4)	0.5467 (3)	0.0644 (11)	
H1A	0.2267	0.1837	0.4874	0.077*	
H1B	0.1607	0.1059	0.5603	0.077*	
N3	0.2713 (4)	0.2314 (4)	0.7073 (3)	0.0660 (11)	
H3A	0.3162	0.2840	0.7540	0.079*	
H3B	0.2139	0.1655	0.7192	0.079*	
C7	0.2905 (5)	0.2520 (4)	0.6168 (3)	0.0552 (11)	
N2	0.3776 (5)	0.3520 (4)	0.5987 (3)	0.0798 (13)	
H2A	0.3884	0.3664	0.5399	0.096*	
H2B	0.4241	0.4034	0.6456	0.096*	
C3	0.1188 (6)	0.4537 (6)	0.3581 (4)	0.0781 (15)	
H3C	0.0303	0.4894	0.3129	0.094*	
H3D	0.0842	0.4046	0.4097	0.094*	
C5	0.2433 (7)	0.7737 (6)	0.4850 (4)	0.0949 (19)	
H5A	0.2832	0.8123	0.4316	0.114*	
H5B	0.1853	0.8444	0.5098	0.114*	
C2	0.2036 (6)	0.3607 (5)	0.3069 (3)	0.0715 (14)	
H2C	0.1349	0.2933	0.2703	0.086*	
H2D	0.2497	0.4123	0.2613	0.086*	
C6	0.4578 (7)	0.8402 (6)	0.5976 (4)	0.0956 (19)	
H6	0.4410	0.9319	0.5799	0.115*	
C4	0.1448 (6)	0.6568 (7)	0.4502 (4)	0.0962 (19)	
H4A	0.1177	0.6107	0.5056	0.115*	
H4B	0.0515	0.6898	0.4082	0.115*	
C1	0.4143 (7)	0.2120 (6)	0.3307 (4)	0.0898 (17)	
H1C	0.4518	0.2667	0.2827	0.108*	
H1D	0.3564	0.1357	0.2969	0.108*	

### Atomic displacement parameters (Å^2^)

**Table:**

	*U*^11^	*U*^22^	*U*^33^	*U*^12^	*U*^13^	*U*^23^
Cl1	0.0803 (9)	0.0601 (7)	0.0605 (8)	−0.0002 (7)	0.0141 (6)	0.0060 (6)
O1	0.068 (2)	0.064 (2)	0.0615 (19)	0.0040 (17)	0.0179 (17)	−0.0044 (16)
O2	0.0586 (19)	0.085 (3)	0.070 (2)	0.0093 (18)	0.0169 (17)	−0.0028 (18)
O3	0.083 (2)	0.068 (2)	0.089 (3)	0.022 (2)	0.006 (2)	−0.0035 (19)
N1	0.078 (3)	0.059 (2)	0.056 (2)	−0.004 (2)	0.014 (2)	−0.003 (2)
N3	0.065 (3)	0.078 (3)	0.055 (2)	−0.007 (2)	0.010 (2)	−0.001 (2)
C7	0.055 (3)	0.049 (3)	0.059 (3)	0.009 (2)	0.008 (2)	0.002 (2)
N2	0.095 (3)	0.075 (3)	0.070 (3)	−0.023 (3)	0.018 (2)	−0.004 (2)
C3	0.061 (3)	0.091 (4)	0.079 (4)	−0.002 (3)	0.009 (3)	0.020 (3)
C5	0.105 (5)	0.091 (5)	0.085 (4)	0.051 (4)	0.011 (4)	−0.005 (3)
C2	0.067 (3)	0.075 (4)	0.064 (3)	−0.016 (3)	−0.004 (3)	0.006 (3)
C6	0.115 (5)	0.050 (3)	0.113 (5)	0.028 (3)	0.003 (4)	−0.011 (3)
C4	0.067 (4)	0.141 (6)	0.082 (4)	0.039 (4)	0.017 (3)	−0.011 (4)
C1	0.102 (4)	0.078 (4)	0.091 (4)	−0.010 (3)	0.024 (4)	−0.034 (3)

### Geometric parameters (Å, º)

**Table:**

O1—C2	1.412 (5)	C3—C2	1.462 (7)
O1—C1	1.418 (6)	C3—H3C	0.9700
O2—C4	1.408 (6)	C3—H3D	0.9700
O2—C3	1.409 (6)	C5—C4	1.459 (8)
O3—C5	1.395 (6)	C5—H5A	0.9700
O3—C6	1.403 (6)	C5—H5B	0.9700
N1—C7	1.319 (5)	C2—H2C	0.9700
N1—H1A	0.8600	C2—H2D	0.9700
N1—H1B	0.8600	C6—C1^i^	1.448 (7)
N3—C7	1.329 (5)	C6—H6	0.9300
N3—H3A	0.8600	C4—H4A	0.9700
N3—H3B	0.8600	C4—H4B	0.9700
C7—N2	1.305 (5)	C1—C6^i^	1.448 (7)
N2—H2A	0.8600	C1—H1C	0.9700
N2—H2B	0.8600	C1—H1D	0.9700
			
C2—O1—C1	112.1 (4)	O3—C5—H5B	109.8
C4—O2—C3	112.7 (4)	C4—C5—H5B	109.8
C5—O3—C6	111.1 (4)	H5A—C5—H5B	108.3
C7—N1—H1A	120.0	O1—C2—C3	109.2 (4)
C7—N1—H1B	120.0	O1—C2—H2C	109.8
H1A—N1—H1B	120.0	C3—C2—H2C	109.8
C7—N3—H3A	120.0	O1—C2—H2D	109.8
C7—N3—H3B	120.0	C3—C2—H2D	109.8
H3A—N3—H3B	120.0	H2C—C2—H2D	108.3
N2—C7—N1	121.6 (4)	O3—C6—C1^i^	108.9 (5)
N2—C7—N3	120.0 (4)	O3—C6—H6	125.6
N1—C7—N3	118.4 (4)	C1^i^—C6—H6	125.6
C7—N2—H2A	120.0	O2—C4—C5	111.9 (5)
C7—N2—H2B	120.0	O2—C4—H4A	109.2
H2A—N2—H2B	120.0	C5—C4—H4A	109.2
O2—C3—C2	108.5 (4)	O2—C4—H4B	109.2
O2—C3—H3C	110.0	C5—C4—H4B	109.2
C2—C3—H3C	110.0	H4A—C4—H4B	107.9
O2—C3—H3D	110.0	O1—C1—C6^i^	110.8 (4)
C2—C3—H3D	110.0	O1—C1—H1C	109.5
H3C—C3—H3D	108.4	C6^i^—C1—H1C	109.5
O3—C5—C4	109.2 (5)	O1—C1—H1D	109.5
O3—C5—H5A	109.8	C6^i^—C1—H1D	109.5
C4—C5—H5A	109.8	H1C—C1—H1D	108.1

Symmetry code: (i) −*x*+1, −*y*+1, −*z*+1.

### Hydrogen-bond geometry (Å, º)

**Table:**

*D*—H···*A*	*D*—H	H···*A*	*D*···*A*	*D*—H···*A*
N1—H1*A*···O1	0.86	2.19	2.976 (5)	152
N1—H1*B*···Cl1	0.86	2.49	3.294 (4)	155
N2—H2*A*···O1	0.86	2.36	3.102 (5)	145
N3—H3*A*···Cl1^ii^	0.86	2.42	3.228 (4)	158

Symmetry code: (ii) −*x*+1/2, *y*+1/2, −*z*+3/2.
